# Determining the Biochemical Properties of the Oxalate Biosynthetic Component (Obc)1 from *Burkholderia mallei*

**DOI:** 10.1371/journal.pone.0163294

**Published:** 2016-09-19

**Authors:** Peter M. Lambert, Paul A. Nakata

**Affiliations:** USDA/ARS Children’s Nutrition Research Center, Department of Pediatrics, Baylor College of Medicine, Houston, TX, 77030–2600; Russian Academy of Medical Sciences, RUSSIAN FEDERATION

## Abstract

Oxalic acid is produced by a variety of organisms ranging from simple microbes to complex animals. This acid has been proposed to fulfill various physiological and pathological functions which vary between organisms. In bacteria from the *Burkholderia* genus, oxalate secretion has been shown to be quorum sensing dependent and to support pathogenicity and cell viability. In light of the critical roles of oxalate in *Burkholderia* as well as other organisms, it is surprising that our understanding of how this simple dicarboxylate is biosynthesized remains incomplete. Here we report the expression, purification, and partial characterization of the first intact bacterial oxalate biosynthetic enzyme, Obc1, from *B*. *mallei*. An N-terminal His-tagged *Bmobc1* was cloned into pDUET, expressed in *E*. *coli* BLR (DE3), and the recombinant enzyme purified by affinity chromatography. Oxalate biosynthetic enzyme assays coupled with HPLC analysis revealed that *Bm*Obc1 catalyzed the biosynthesis of oxalate, acetoacetate, and free CoA from oxaloacetate and a short chain acyl-CoA following Michaelis-Menten kinetics. Optimal enzyme activity was measured at pH 8.0 and a temperature around 44°C. Kinetic analysis conducted under conditions of saturating acetyl-CoA and varying oxaloacetate concentrations resulted in a calculated Km value for oxaloacetate of 94.3± 9.2 μM (mean ± SE). Under conditions of saturating oxaloacetate concentration and varying acyl-CoA (acetyl- or propionyl-CoA) concentrations kinetic analysis generated a calculated Km value of 26.8 ± 2.3 μM (mean ± SE) for acetyl-CoA and 104.4 ± 12.7 μM for propionyl-CoA. The significantly lower K_m_ for acetyl-CoA suggests that it is strongly favored as a substrate over propionyl-CoA.

## Introduction

Oxalate, the smallest dicarboxylic acid, is synthesized by a variety of organisms, including bacteria, fungi, plants, and some mammals [1]. Oxalate has been proposed to have many different functions which, along with its chemical form and distribution, vary among organisms (reviewed by[1–4]). In microorganisms, oxalate has been shown to protect against metal toxicity [2, 5, 6], act as a pathogenicity factor [7–12], and enable nutrient acquisition [2, 13–15]. In addition, it was recently reported that oxalate biosynthesis was required by bacteria from the *Burkholderia* genus to maintain cell viability through its role in neutralizing the toxic effects of ammonia accumulation [16]. *Burkholderia* mutants impaired in oxalate biosynthesis or in the quorum sensing pathway preceding the biosynthesis of the acid were unable to maintain stationary-phase growth and underwent a sudden collapse in population [16]. Exogenous neutralization of this base toxicity restored cell viability signifying the importance of oxalate biosynthesis to the survival of the bacterial population [16].

This central role for oxalate biosynthesis in maintaining cell viability coupled with its identified role as a pathogenicity factor in both fungi [7, 8, 10–12] and bacteria [9] suggests that oxalate biosynthesis may be a viable target for the development of a new strategy to combat oxalate-secreting pathogens. In support of this strategy, studies have shown that plants engineered to over-express oxalate catabolizing enzymes to degrade this pathogenicity factor have increased resistance to oxalate-secreting phytopathogens [17–21]. Thus, development of a strategy to inhibit oxalate biosynthesis is likely to benefit efforts to combat a variety of fungal and bacterial pathogens including a number of animal and plant pathogens from within the *Burkholderia* genus [9, 22–24]. Economically important plant pathogens from this genus include *B*. *glumae* which is a broad-range pathogen responsible for ravaging a number of crop plants including rice which is a staple food for many around the world [9, 22, 25]. Animal pathogens from this genus include *B*. *mallei* [26] and *B*. *pseudomallei* [27] both of which are listed as potential biological warfare agents [28], by the Center for Disease Control and Prevention, as a result of their highly infectious nature. Although *B*. *mallei* is an obligate animal pathogen that typically infects horses, mules, and donkeys it is also capable of infecting humans. Once infected, even with proper antibiotic treatment, the mortality rate for humans is 50 percent [29, 30]. Due to the lack of an effective treatment for *B*. *mallei* infections, and its potential use as a biological warfare agent, it is important to be proactive in the search for new strategies to combat such an oxalate-secreting pathogen.

Before rational strategies can be designed to specifically target oxalate-secreting pathogens, such as *B*. *mallei*, a better understanding of how these organisms biosynthesize oxalate is required. In *Burkholderia*, an effort to gain this understanding was first made by attempting to identify the oxalate biosynthetic enzyme from the plant pathogen, *B*. *glumae* [9]. Although difficulties were encountered in purification of the biosynthetic activity, crucial information was reported. Such information included the development of an assay to measure oxalate biosynthetic activity. Difficulties in purification of this activity could be explained, at least in part, upon discovery that this biosynthetic activity from *B*. *glumae* was encoded by two genes, *oxalate biosynthetic component (obc)A* and *obcB* [31]. Attempts to purify the recombinant oxalate biosynthetic enzyme through co-expression of ObcA and ObcB have been hampered by insolubility issues with ObcB [32]. Thus, structural and functional characterization of this biosynthetic activity has thus far focused on ObcA which catalyzes the first-step in a two-step sequential reaction of oxalate biosynthesis [32]. In contrast to *B*. *glumae*, the oxalate biosynthetic activity in *B*. *mallei*, as well a number of the other *Burkholderia* species, was found to be encoded by a single gene locus named *obc1* [33]. Although Obc1 had a predicted protein length of 1125 amino acids compared to the 540 amino acid length of ObcA, the two proteins still shared a 50% overall amino acid identity [32, 33]. The homology between the two proteins; however, was restricted to the N-terminal portion of Obc1 [32, 33]. No significant homology was found between the C-terminal portion of Obc1 and ObcA or ObcB [33]. The possibility that Obc1 encoded an oxalate biosynthetic activity more amenable to recombinant expression and purification warranted further investigation.

As a step toward elucidating the biochemical properties of an intact oxalate biosynthetic enzyme from bacteria we first constructed and expressed an N-terminal His-tag *B*. *mallei* Obc1 fusion protein in *E*.*coli*. The enzyme was found to be soluble allowing its purification by affinity chromatography. Enzyme assays showed that purified His-*Bm*Obc1 catalyzed the biosynthesis of oxalate from oxaloacetate and a short-chain acyl-CoA. Substrate specificity and product analysis showed that this enzyme, encoded by a single gene, functioned in a manner similar to the *B*. *glumae* enzyme which is encoded by two genes. This finding suggests that different members of the *Burkholderia* genus may utilize the same pathway of oxalate biosynthesis regardless of the number of genes required to encode the biosynthetic activity.

## Materials and Methods

### Media, strains, and reagents

*Escherichia coli* (DH5α, Invitrogen Life Technology, Carlsbad, CA; BLR (DE3), EMD Biosciences, Madison, WI) were grown in Luria Broth (Invitrogen Life Technology) media. Organic acids, acyl-CoAs, antibiotics, and oligonucleotide primers were purchased from Sigma-Aldrich (St. Louis, MO). Restriction enzymes were bought from New England Biolabs (Ipswich, MA).

### Cloning and Expression of His-*Bmobc1*

Primers were designed from the genomic sequence of *B*. *mallei* [26, 33] to amplify *Bmobc1* using PCR. Addition of an N-terminal poly-histidine tag was engineered with gene specific primers 5’-TTCATATGGGCAGCAGCCATCACCATCATCACCACATGGTCAA GACCTTCTACATCAC-3’ and 5’-TGGTACCGATCGGGTTACGCGAGTGCTCG-3’. An engineered *Nde*I site was included in the 5’-primer, and the 3’-primer contained an engineered *Kpn*I site. The PCR reaction was conducted using the PCRx Enhancer kit (Invitrogen Life Technologies) according to the manufacturer’s instructions, and the amplified gene containing the poly-histidine tag was cloned with the Qiagen TA cloning kit (Qiagen Inc., Valencia, CA). *His-Bmobc1* was then subcloned unidirectionally into the pDUET vector (Novagen, EMD Biosciences, Inc., Madison, WI) that was digested with *Nde*I and *Kpn*I.

The pDUET Expression vector containing *His-Bmobc1* was transformed into *E*. *coli* strain BLR(DE3) for recombinant enzyme growth. A starter culture was grown overnight at 37°C with shaking and diluted 1:100 in LB media supplemented with 50 μg/ml carbenicillin and incubated at 37°C with shaking until it reached an OD_600_ of 0.8. Expression was induced by the addition of IPTG to a final concentration of 1mM, and the culture was allowed to grow for 4h at 30°C with shaking. The cells were collected by centrifugation and stored at -70°C until lysed for protein purification.

### Purification of His-*Bm*Obc1

Cell lysis and protein purification was performed according to the manufacturer’s protocol for purification under native conditions described in the Ni-NTA buffer kit from Novagen (EMD Millipore, Billerica Massachusetts). Frozen cells were thawed on ice for 15 min and resuspended in binding buffer (50 mM NaH_2_ PO_4_, pH 8.0; 300 mM NaCl; 10 mM imidazole) supplemented with lysozyme (10 mg/ml) and benzonase (Novagen). After 20 min of incubation on ice, the cells were sonicated and centrifuged at 27,000 x *g* for 20 min at 4°C. The supernatant was collected and loaded onto a column packed with PerfectPro Ni-NTA agarose (5Prime, Gaithersburg, MD) that had been equilibrated with binding buffer. After allowing the loaded supernatant to drain from the column, the resin was washed first with binding buffer, followed by wash buffer (50 mM NaH_2_ PO_4_, pH 8.0; 300 mM NaCl; 20 mM imidazole). Bound protein was eluted in 6 column volumes of elution buffer (50 mM NaH_2_ PO_4_, pH 8.0; 300 mM NaCl; 250 mM imidazole). The eluted protein was concentrated with an Amicon Ultra 30K device (EMD Millipore, Billerica Massachusetts) and the buffer exchanged to 50 mM Tris-HCl, pH 8.0; 100 mM NaCl; 10% (w/v) sucrose. Purified protein was flash frozen in liquid nitrogen and stored at -70°C until use. The protein measurements were determined by Bradford assay (Bradford, 1976). Protein purity was assessed by SDS-PAGE and the proteins visualized by Coomassie Brilliant Blue R 250 staining (Biorad, Hercules, CA).

### Assessment of *Bm*Obc1 Substrate Specificity

To investigate the substrate specificity of *Bm*Obc1, various organic acids and short-chain acyl-CoAs were used in a modified enzymatic assay [9] where 1000 μM glyoxalic, lactic, succinic, malic, or citric acid was substituted for oxaloacetate. Additionally, 500 μM propionyl-CoA or butyryl-CoA was substituted for acetyl-CoA. The reactions were allowed to run for 30 min and the production of oxalic acid determined as described below.

### Oxalate Determinations

Oxalate quantification was performed with an oxalate diagnostic kit (Trinity Biotech, St. Louis, MO) according to the manufacturer’s instructions as previously described [33]. In brief, the oxalate was oxidized by oxalate oxidase to carbon dioxide and hydrogen peroxide. The generated hydrogen peroxide was then allowed to react with 3-methyl-2-benzothiazolinone hydrazone and 3-(dimethylamino) benzoic acid in the presence of peroxidase to give an indamine dye that was read at 590 nm using a SpectraMax M2 plate reader (Molecular Devices, Sunnyvale, CA). Oxalate measurements were performed in triplicate. In addition, oxalate values determined via the oxalate kit were verified by HPLC according to Foster and Nakata [34].

### pH and Temperature Optimization of *Bm*Obc1

To determine the optimum pH for His-*Bm*Obc1 activity, purified *Bm*Obc1was added to modified assay system 2 [9] containing 1000 μM oxaloacetic acid, 400 μM acetyl-CoA, 100 mM NaCl, and 100 μM CoCl_2_ in either 50 mM Tris-HCl, pH 6.0–8.0, or 50 mM NaPO_4_, pH 7.5–9.0. The reaction was carried out for 5 min and the samples were flash frozen in liquid nitrogen and stored at -70°C until the oxalate produced by the reaction was quantified as described above. The experiment was performed three times with each measurement conducted in duplicate.

For temperature optimum determination, the modified assay was performed in 50 mM NaPO_4_, pH 8.0 at temperatures varying from 4–75°C. The reaction mixture was equilibrated at the assay temperature for 2 min before the addition of purified *Bm*Obc1. After 5 min, the samples were flash frozen and stored at -70°C until oxalate determinations were made. Care was taken to quantify the oxalate immediately after thawing. All samples were thawed at the same temperature for the same amount of time and then diluted 1:20 to minimize any oxalate biosynthesis that might occur during the detection process. Oxalate production was represented as relative activity as an additional safeguard. Assays at each temperature were performed three times and each measurement conducted in duplicate.

### Steady State Kinetics of *Bm*Obc1

A steady-state kinetic analysis of *Bm*Obc1 was performed by monitoring the production of free CoA over time as previously described [32]. In brief, the decrease in absorbance of 2,6-dichlorophenolindophenol (DCPIP), a dye that reacts with the sulfhydryl group on CoA, was monitored by spectrophotometry at 600 nm. The basic reaction mixture consisted of 50 mM NaPO_4_ (pH 8.0), 100 mM NaCl, 100 μM CoCl_2_, and 100 μM DCPIP. A range of substrate concentrations were added to this basic reaction mixture. Acyl-CoA (either acetyl- or propionyl-CoA) was varied from 10–500 μM under saturating concentrations of oxaloacetate (1000 μM) and oxaloacetate concentrations varied from 10–1000 μM under saturating acyl-CoA (acetyl- or propionyl-) concentrations. The assay was initiated by the addition of 10 μg purified *Bm*Obc1 and the reaction was monitored for 3 min. The reaction velocity was determined from the linear decrease in absorbance observed after 120 secs from the start of the assay. The assays were performed in duplicate and the experiments were performed in triplicate. The data were fit to the Michaelis-Menten model using Prism 6.0 (GraphPad) and the K_m_ and V_max_ for each substrate were determined.

### Identification of Reaction Products

The products of the *Bm*Obc1 enzymatic reaction were determined using a slightly modified assay system 2 [9] in which Hepes buffer was replaced by 50 mM NaPO_4_, pH 8.0. An aliquot of the reaction mix was taken before the addition of purified *Bm*Obc1, and additional aliquots were taken 10 and 30 min after the reaction was initiated. The reaction was stopped by separating the enzyme from the assay mixture with the use of an Amicon Ultra 30K centrifugal device (Merck Millipore Ltd, Billerica, MA). Reaction products were analyzed by HPLC as previously described [35].

In brief, CoA compounds were resolved using an Agilent 1100 HPLC (Agilent Technologies, Santa Clara, CA) coupled to a photodiode array detector (Agilent 1100) at 254 nm with a C-18 reversed-phase Synergi 4μ Hydro-RP 80A, 250 x 4.6 mm column (Phenomenex, Torrance, CA) equilibrated with 86% buffer A (25 mM, NaOAc, pH 4.5) and 14% buffer B (20 mM, NaOAc, pH 4.5, 20% CH_3_CN) at 0.75 mL/min. Following the injection of 15μL of the *Bm*Obc1 reaction mixture, a 15 minute linear gradient to 40% buffer B was initiated, followed by a step to 100% buffer B for two minutes, then a step back to 14% buffer B for the remaining time of the 28 min run. The identity of each eluted compound was validated by comparison to commercially available external standards (Sigma-Aldrich, St. Louis, MO). Carboxylates were resolved on the Synergi C-18 column equilibrated in 100% buffer A at 0.75 mL/min at 230 nm. After the injection of 40μL of the *Bm*Obc1 reaction mixture, the acids were separated with an 8 min isocratic elution in 100% buffer A, followed by a step to 40% buffer B and a four min linear gradient to 100% buffer B. The column was washed for 2 min at 100% buffer B before a step to 100% buffer A for the remainder of the 26 min run. Peak identity was determined by comparison to prepared standards (Sigma-Aldrich).

## Results and Discussion

Although oxalic acid is common in nature and has a broad impact on a host of organisms our understanding of how these organisms biosynthesize this acid is still incomplete. As a step toward expanding our understanding of bacterial oxalate biosynthesis we define the enzymatic reaction and characterize the kinetic parameters of the Obc1 from *B*. *mallei*.

### Defining substrate specificities of *Bm*Obc1

**Fig 1:**
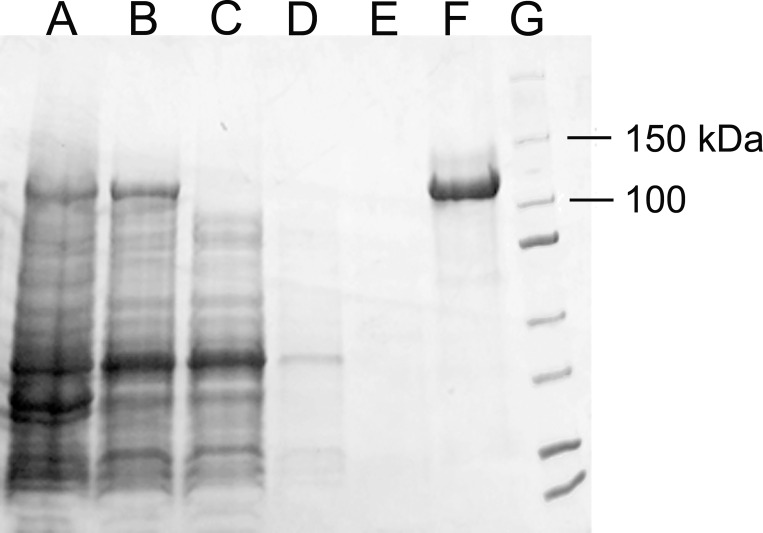
Expression and Purification of *Bm*Obc1. SDS-PAGE gel of nickel-affinity purified *Bm*Obc1 protein stained with Coomassie blue showing >90% purity. Aliquots from each step of the purification process were fractionated onto a 4–15% acrylamide gel. Lane (A) crude extract; (B) cleared lysate; (C) flow through; (D) wash 1 (E) wash 2; (F) eluted fraction; (G) protein standards

To investigate the substrate specificity of *Bm*Obc1, we cloned the His-tagged-fusion of the enzyme into the pDUET vector, expressed the construct in *E*. *coli*, and purified the recombinant enzyme by nickel-affinity chromatography. Based on the fractionation profiles generated by SDS-PAGE, this purification procedure resulted in a BmObc1 fraction that was estimated to be about 90% pure (Fig 1). A spectrophotometric assay was used to assess the BmObc1 catalyzed conversion of different carbon chain length dicarboxylic acids, in the presence of acyl-CoAs, into oxalic acid (Table 1). Oxalic acid was detected in the reaction mixes containing the four carbon dicarboxylate, oxaloacetate, and the 2 or 3 carbon short chain acyl-CoAs, acetyl-CoA and propionyl-CoA, respectively (Table 1). No oxalate production was detected from oxaloacetate in the presence of an acyl-CoA longer than 3 carbons nor did any of the other dicarboxylate lead to the production of the acid in the presence of any of the listed acyl-CoA (Table 1). Overall, the substrate specificity of the recombinant *Bm*Obc1 appeared similar to the specificities determined for the *B*. *glumae* biosynthetic activity using partially purified extracts [9].

**Table 1:** Substrate specificity of *Bm*Obc1.

		Acetyl CoA CH_3_CO-CoA	Propionyl-CoA CH_3_CH_2_CO-CoA	Butyryl-CoA CH_3_CH_2_CH_2_CO-CoA
Glyoxylic Acid	CHO-COOH	-	-	-
Lactic Acid	CH_3_CHOH-COOH	-	-	-
Succinic Acid	HOOC-CH_2_CH_2_CHOH-COOH	-	-	-
Malic Acid	HOOC-CH_2_CHOH-COOH	-	-	-
Oxaloacetic Acid	HOOC-COCH_2_-COOH	+	+	-
Citric Acid	HOOC-CH_2_COHCOOHCH_2_-COOH	-	-	-
+/- Production of Oxalic Acid				

### Defining optimal *Bm*Obc1 reaction conditions

**Fig 2:**
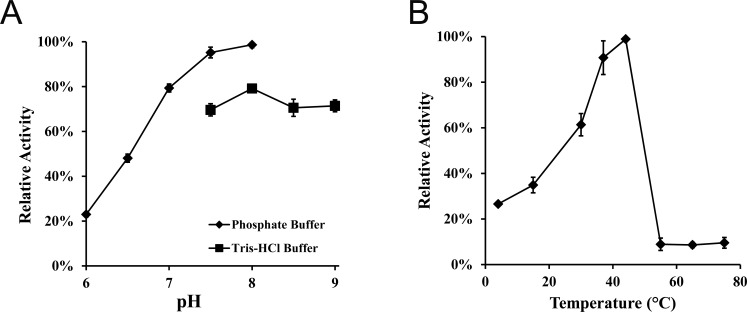
Determination of optimal *Bm*Obc1 reaction conditions. (A) The optimum pH for *Bm*Obc1 activity was determined *in vitro* by measuring the amount of oxalate produced over five minutes using 1000 μM oxaloacetic acid (OAA) and 400 μM acetyl-CoA. Two buffer systems were utilized to cover this broad pH range: sodium phosphate for pH 6.0 to 8.0, and Tris-HCl for pH 7.5 to 9.0. Data are mean ± SE for three trials of duplicate assays. (B) For optimal temperature determination, assays were performed at temperatures ranging from 4–75°C in sodium phosphate buffer, pH 8.0. Data are mean ± SE of three trials.

The *Bm*Obc1 showed *in vitro* biosynthetic activity over a wide pH range with an optimum at a pH of 8.0 (Fig 2A). Although a second pH optimum outside of the tested range cannot be completely ruled out, the measured pH optimum of 8 suggests that it could be beneficial to the bacterial population to produce oxalic acid when the pH becomes alkaline. This notion is consistent with a recent study that has shown that several species of *Burkholderia* exhibited a collapse in population when the culture media pH rose above 8.0 [16]. This increase in alkalinity has been attributed to the accumulation of ammonia that is excreted by the growing bacterial population [16].

The optimal temperature for His-*Bm*Obc1 was also determined (Fig 2B). The activity of His-*Bm*Obc1 increased steadily with increasing temperature from 4°C up to 44°C. A dramatic decrease to <15% relative activity occurred once temperatures reached 55°C or above. The optimal temperature for the production of oxalate by *Bm*Obc1 was approximated to fall between 37–44°C. The body temperature of a horse, the natural host for *B*. *mallei*, is around 38°C and would rise in a fever response to infection. Thus the optimal temperature for *Bm*Obc1 may allow oxalate to be produced and the bacterial population to prosper in its host environment under normal and elevated body temperatures.

### Defining the steady-state kinetics of *Bm*Obc1

**Fig 3:**
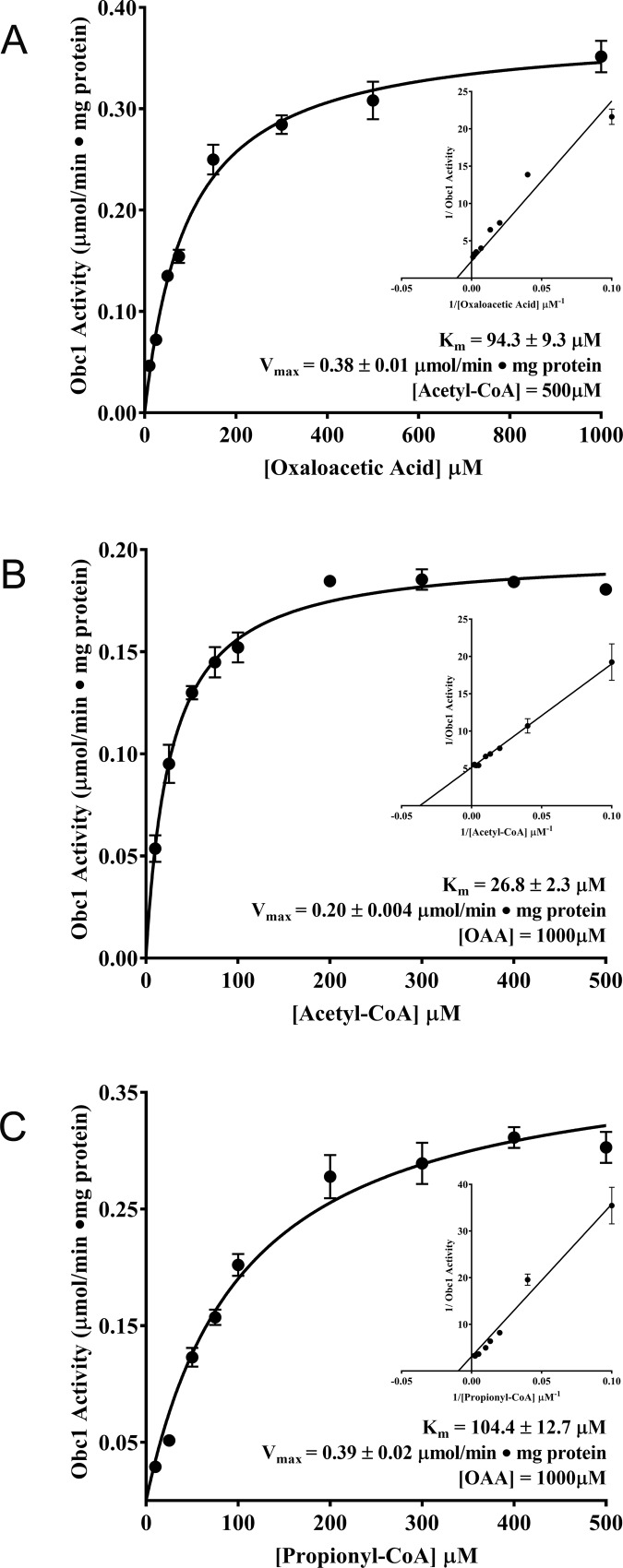
Steady State Kinetics of *Bm*Obc1. Kinetic analysis of *Bm*Obc1 was performed over a range of substrate concentrations. (A) Kinetic analysis of *Bm*Obc1 under saturating acetyl-CoA concentrations and oxaloacetic acid (OAA) concentrations ranging from 10 to 1000μM. (B) Kinetic analysis of *Bm*Obc1 under saturating oxaloacetic acid concentrations and acetyl-CoA concentrations ranging from 10 to 500μM. (C) Kinetic analysis of *Bm*Obc1 under saturating oxaloacetate concentrations and propionyl-CoA concentrations ranging from 10 to 500μM. K_m_ values were calculated using non-linear regression to the Michaelis-Menten model. Data are mean ± SE from at least three experiments

*In vitro* enzyme assays revealed that *Bm*Obc1 exhibited Michealis-Menten kinetics with respect to both oxaloacetate and short-chain acyl-CoAs (Fig 3). The *Bm*Obc1 kinetic parameters for each substrate were determined from a nonlinear regression to the Michaelis-Menten model. A K_m_ value of 94.3 ± 9.2 μM was calculated for oxaloacetate by conducting enzyme assays using reaction mixtures containing varying oxaloacetate concentrations and saturating acetyl-CoA concentrations (Fig 3A). Enzyme assays conducted under conditions of saturating oxaloacetate concentration and variable acyl-CoA concentrations resulted in a calculated K_m_ value of 26.8 ± 2.3 μM (mean ± SE) for acetyl-CoA (Fig 3B) and 104.4 ± 12.7 μM for propionyl-CoA (Fig 3C). The significantly lower K_m_ of acetyl-CoA suggests that this two carbon acyl-CoA is strongly favored as a substrate over the three carbon propionyl-CoA. This preference could be a consequence of the shorter acyl chain having easier access to the active site of *Bm*Obc1.

In comparison to the substrate affinities of ObcA, *Bm*Obc1 showed higher affinity for both substrates, but the difference in affinity for acetyl-CoA was most notable with the reported Km value of 58.6 ± 12.8 for ObcA [32]. This suggests that *B*. *mallei* is capable of effectively producing oxalate at much lower cellular acetyl-CoA concentrations than *B*. *glumae*. The significantly higher affinity for acetyl-CoA than oxaloacetic acid observed for *Bm*Obc1 is also consistent with what was reported for ObcA from *B*. *glumae* [32]. Therefore, in both species of *Burkholderia*, oxalate biosynthetic activity appears to be more sensitive to decreases in the relative availability of oxaloacetate than acetyl-CoA. Whether the oxaloacetate and the acetyl-CoA bind and react similarly in the active site of the *B*. *mallei* and *B*. *glumae* enzymes remains unknown. However, the ability of the N-terminal fragment of *Bm*Obc1 to substitute for ObcA, coupled with the conservation of residues in *Bm*Obc1 identified as necessary for ligand binding and catalysis in ObcA suggests that at least the initial mechanism for combining acetyl-CoA and oxaloacetate is similar in both species [32, 33].

### Defining the *Bm*Obc1 oxalate biosynthetic reaction

**Fig 4:**
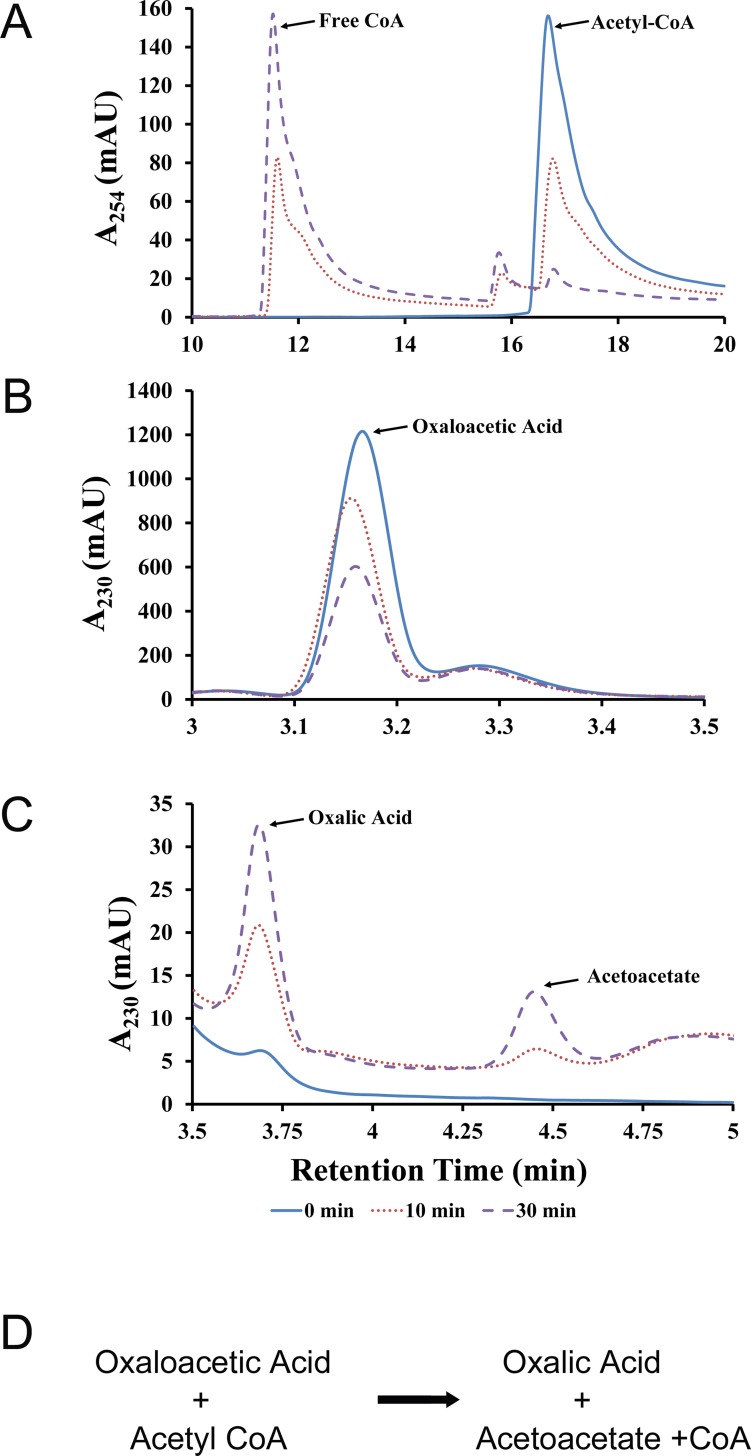
Defining the *Bm*Obc1 enzyme reaction. C-18 Reverse-Phased HPLC was utilized to separate and identify the compounds by comparison to known standards. The traces show the conversion of oxaloacetic acid and acetyl-CoA to oxalic acid, acetoacetate, and free CoA. Note the correlation between decrease in substrates and increase in products over time. (A) The acetyl-CoA and free CoA were monitored at 254 nm. (B) Oxaloacetic acid was monitored at 230 nm. (C) Oxalic acid and Acetoacetate were detected at 230 nm. (D) Derived oxalic acid biosynthetic reaction catalyzed by *Bm*Obc1.

HPLC analysis was employed to further define the *Bm*Obc1 catalyzed oxalate biosynthetic reaction in terms of the generated reaction products (Fig 4). Purified *Bm*Obc1 was added to a reaction mix containing the substrates acetyl-CoA (Fig 4A) and oxaloacetate (Fig 4B). As expected, the amount of each substrate was observed to decrease as the reaction progressed. This reduction in substrates correlated with an increase in the production of three compounds which were identified by comparison to known standards as free CoA, oxalic acid, and acetoacetate (Fig 4C). This comparative analysis showed that *Bm*Obc1 catalyzed the conversion of oxaloacetate and acetyl-CoA to oxalate, acetoacetate, and free CoA (Fig 4D) which is in agreement with the oxalate biosynthetic reaction products reported in a study of the *B*. *glumae* oxalate biosynthetic activity [9].

## Conclusion

In this study we initiated the characterization of the biosynthetic properties of the *Bm*Obc1 enzyme from *B*. *mallei*. Expanding our understanding of the biochemical properties of this enzyme may prove useful in efforts to develop a new strategy to combat oxalate-secreting pathogens through inhibition of oxalate biosynthesis. Analysis of substrate specificities and reaction products indicated that both animal and plant pathogens from the *Burkholderia* genus utilized the same enzymatic reaction to biosynthesize oxalic acid, irrespective of the number genes required to encode the biosynthetic activity. This common link indicates that a single inhibitory strategy may be applicable to a variety of host pathogens at least within the *Burkholderia* genus.
